# The Extension of Phase Correlation to Image Perspective Distortions Based on Particle Swarm Optimization

**DOI:** 10.3390/s19143117

**Published:** 2019-07-15

**Authors:** Xue Wan, Chenhui Wang, Shengyang Li

**Affiliations:** 1 Technology and Engineering Center for Space Utilization, Chinese Academy of Sciences, Beijing 100094, China; 2 Key Laboratory of Space Utilization, Chinese Academy of Sciences, Beijing 100094, China; 3 School of Remote Sensing and Information Engineering, Wuhan University, Wuhan 430079, China

**Keywords:** phase correlation, perspective, optimization

## Abstract

Phase correlation is one of the widely used image registration method in medical image processing and remote sensing. One of the main limitations of the phase correlation-based registration method is that it can only cope with Euclidean transformations, such as translation, rotation and scale, which constrain its application in wider fields, such as multi-view image matching, image-based navigation, etc. In this paper, we extended the phase correlation to perspective transformation by the combination of particle swarm optimization. Inspired by optic lens alignment based on interference, we propose to use the quality of PC fringes as the similarity, and then the aim of registration is to search for the optimized geometric transformation operator, which obtain the maximize value of PC-based similarity function through particle swarm optimization approach. The proposed method is validated by image registration experiments using simulated terrain shading, texture and natural landscape images containing different challenges, including illumination variation, lack of texture, motion blur, occlusion and geometric distortions. Further, image-based navigation experiments are carried out to demonstrate that the proposed method is able to correctly recover the trajectory of camera using multimodal target and reference image. Even under great radiometric and geometric distortions, the proposed method is able to achieve 0.1 sub-pixel matching accuracy on average while other methods fail to find the correspondence.

## 1. Introduction

Image matching is one of the fundamental problems in the fields of computer vision and photogrammetry. The applications of image matching are various, including camera calibration [1], 3D reconstruction [2], visual navigation [3], super-resolution [4], etc. Many image matching algorithms have been proposed to accomplish the above tasks. Among them, feature-based methods, which focus on low-level features, such as edges, corners, are the most widely applied. Although many feature-based matching algorithms have been proved to achieve robust image matching between image pairs, they largely rely on the abundance of image texture, and may fail in the cases of lack of texture or repetitive texture. The problem is that by dividing an image into several small matching windows, or image patches, the overall structural similarity has been neglected. Most feature-based methods basically rely on low-level features, ignoring higher level features. 

Instead of attempting to match local image features, correlation-based methods match the total or an area of image texture [5]. Compared to feature-based methods, correlation-based methods are able to take the overall structural similarity into consideration, so that they are proved to be more robust to a lack of texture images when limited features can be extracted in local areas. Phase correlation [6], as one of the widely used correlation-based image matching algorithms, has proved to be able to cope with repetitive texture and appearance differences between multimodal image pairs [7]. However, correlation-based methods can hardly cope with large image distortion, which prevents them from further application in image registration under complex geometric transformation, such as perspective, affine, polynomial, etc. They are mainly used in dense matching using small matching windows that can be considered as a simple image translation. 

**Figure 1:**
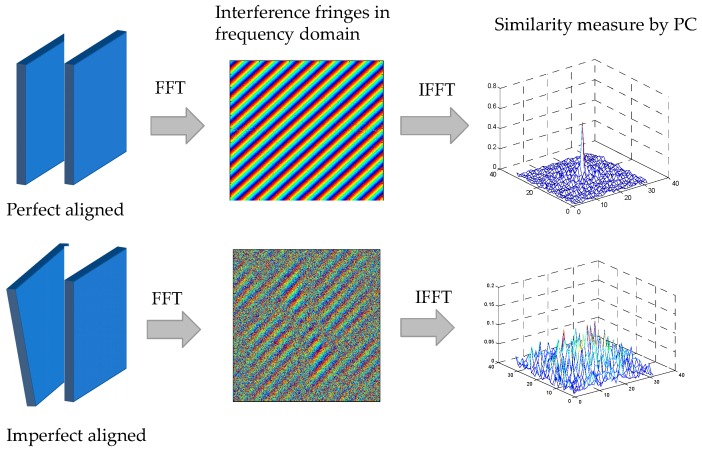
Interference-based lens alignment based on phase correlation.

In this paper, we extend phase correlation to complex geometric distortions by taking the advantage of its sensitivity to geometric distortion as the indicator for optimization. This work is particularly motivated by optic lens alignment based on interference. In interferometric optical testing, interference fringes are indicators to determine whether two lens are perfectly aligned or not. In this paper, we mimic the processor of interference-based lens alignment using novel optimization-based technique. If two images are perfectly aligned, the PC cross-power spectrum of the images taken by the two cameras appear to have clear fringe pattern, and the Dirac delta function which is the IFT of the cross-power spectrum will have a distinctive peak, as shown in Figure 1. Otherwise, the fringe patterns will become vague, and the peak value are not distinctive. The best alignment is achieved by iteratively altering the geometric parameters of the camera until the interference fringes are strongest and clear.

This paper is organized as follows: the related work is reviewed in Section 2. In Section 3, we present our extension of the phase correlation to perspective geometric distortion. Experiment results are then given in Section 4 from image registration to image-based navigation using DEM simulated terrain shading images as well as true landscape images with comparison to state-of-the art image matching methods. The paper is concluded in Section 5.

## 2. Related Work

Image registration methods proposed thus far are generally either feature-based or correlation-based. The foundation of feature-based image matching is under the assumption that distinctive features maintain their positions and shape geometry under different imaging conditions. Most corner and edge detection algorithms are based on the spatial domain, which detect the sharply changed patterns based on image brightness. Some commonly used edge detection algorithms include the Canny operator [8], zero-crossing operator [9] and Mar operator [10], and more recently, Scale-invariant Feature Transform (SIFT) [11], proposed by Lowe. SIFT is invariant to scale, rotation and illumination intensity change, which enables robust image matching. Several improvements have been made to the SIFT operator to enhance its robustness for matching. One of the most widely accepted improved algorithms is Speed Up Robust Features (SURF) [12], which claims to be faster and more robust than SIFT. These hand-craft features have achieved a dominant position in computer vision for years for their flexibility to different geometric distortions. 

Recently, Convolution Neural Network (CNN)-based methods have been widely used in computer vision, including object detection, image retrieval, etc. Some of the studies applied learning-based approach for feature-based image matching. Temporally Invariant Learned Detector (TILDE) [13] uses piecewise linear convolution filters to train data to robustly detect feature points which are robust to illumination and seasonal variations. Yi, et al. [14] proposed a Learned Invariant Feature Transform (LIFT) algorithm which is an end-end deep network including feature detection, orientation estimation and feature description. To narrowing the gap between training data and testing data, a self-supervised interest point detector has been proposed [15] which pre-trained the feature extractor on MS-COCO generic images. By using homographic adaptation approach, this fully-convolutional model achieves superior matching performance compared to state-of-the-art feature matching algorithms. The main limitation of deep learning-based approach is the requirement of large-scale annotated training data. To generate such large-scale training data which contain most cases in real-world is non-trivial. 

The majority of feature-based matching methods solve perspective image distortion problems based on the following assumption: although the transformations between image pairs are rather complex, the transformations between small matching windows can be simplified as image translation or Euclidean transformations, which include translation, scale and rotation. Then, a complex geometric transformation matrix, such as affine and projective, can be estimated by fitting all the feature points that do not contain outliers. Thus, as shown in Table 1, for the image pairs lacking abundant texture or from multimodal data, few features could be extracted which may lead to incorrect transformation matrix estimations.

Correlation-based image matching approaches, known as area-based matching, are another branch of image matching approach. Area correlation based algorithms, such as Normalised Cross Correlation (NCC), Mutual Information (MI) [16] and Phase Correlation (PC) [6], directly match two areas in the reference and target images without feature extraction. Phase Correlation is an image matching algorithm based on Fourier shift property, which states that a translation shift between two similar images generates a linear phase difference in the Fourier frequency domain [6]. The image shift can be resolved directly in the frequency domain with sub-pixel accuracy by unwarping and rank one fitting of the fringe patterns in the cross power spectrum [17,18,19,20]. The rotation and scale differences between matched images for matching can also be estimated by transferring the Fourier spectra to a log-polar plane after applying a high pass filter [21]. Our previous work [22] demonstrated that the robustness of PC to illumination change via theoretical proof and experiment results. 

Compared to feature-based algorithms, area based matching algorithms are usually dependens on the global grey value distribution and thus are more robust to lack-of texture and multimodal. One disadvantage of correlation-based approaches is that they cannot tolerate complex geometric distortions, because the similarity measurement is based on windows [23]. Thus, correlation-based approaches are not applicable to direct registration between two images which have large view angle difference, as shown in Table 1.

For robust image matching, an algorithm needs to be robust to radiometric distortions, such as lack-of texture and multimodal, and robust to large geometric distortions as denoted in the last row of Table 1. Studies thus far have shown that phase correlation matching can achieve sub-pixel accuracy in estimation of translation, rotation, scale and illumination changes between matched images and it is robust to random noise [21]. However, how to enhance the robustness of PC to perspective distortions remains unsolved. Effendi and Jarvis [24] estimated camera ego-motion using phase correlation, however, this method requires a plane, such as table plane, to be discovered in images. Other studies [25,26] apply phase correlation using small matching window and then estimated the geometric transformation based on corresponding result from phase correlation matching. These approaches have the similar limitation to most feature-based methods as they are based on local grey value distribution while regardless of overall structural similarity. Moreover, the approach cannot cope with large view angle difference, because if phase correlation is not robust to perspective distortions, the correspondence within small matching window cannot be found by phase correlation. In this paper, we propose a Phase Correlation-based iterative matching approach to solve large geometric distortion problem and achieve sub-pixel matching accuracy in some challenging image matching cases, such as lack of texture, occlusion, motion blur and multi-modal. Particularly, the contributions of this work are summarized as follows:

- We extend the phase correlation, which only cope with translation, scale and rotation, to perspective variation by the combination of particle swarm optimization (PSO).
- The Dirac delta function in Phase Correlation has been proposed as measure similarity degree for optimization to determine the rotation parameter.
- The proposed method can solve different geometric distortions includes affine, perspective, etc.

## 3. Optimization-Based Phase Correlation

The aim of optimization based registration is to search for the optimized transformation operator $G_{opt}$, which obtain the maximum value of similarity function *S* through certain optimization approach [27]:

(1) $$G_{opt}:I_{1}\to I_{2}=maximize\left(S\left(I_{1}-G\left(I_{2}\right)\right)\right)$$

where G represents the transformation operators.

Optimization algorithms can be divided into two categories: analytical methods and heuristic methods. The analytical optimization approach solves the differential in an objective function. One of the commonly used methods in optimization based on continuous variable is gradient descent (GD), that has been used in solving several image registration problems [28,29]. Compared to analytical optimization, heuristic optimization methods are based on Markov random field, and have the advantage of high calculation efficiency [27]. Thus, it has been used to solve the complex large-scale optimization problems [28,29].

**Figure 2:**
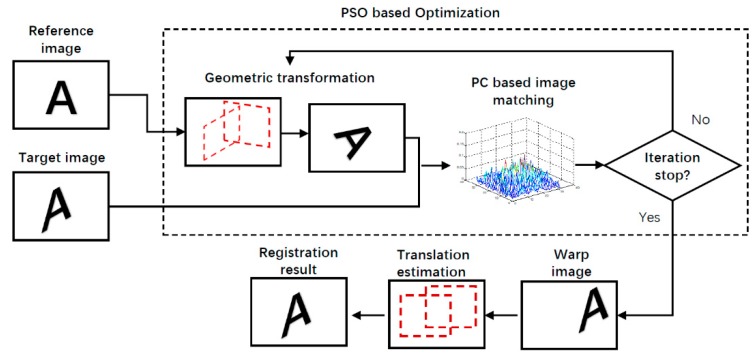
Pipeline of the proposed image registration which combine phase correlation and particle swarm optimization.

This paper proposes a discrete variable optimization approach based on a phase correlation matching algorithm, which is shown in Figure 2. We firstly decompose geometric transformations into rotations and translations. The rotation parameters are determined by optimization using phase correlation as similarity measure and translation parameters are determined by directly by phase correlation. There are four main steps in the proposed matching algorithm:

(1) Determine the transformation type between the target and reference images
(2) Calculate the similarity between images in frequency domain using the Dirac delta function in Phase Correlation
(3) Find the rotation transformation parameters to reach the optimal similarity value based on Particle Swarm optimization.
(4) Calculate the translation parameters using phase correlation

The geometric transformation function will be detailed described in Section 3.1. The similarity function *S* is based on the frequency similarity presenting by phase correlation, which will be described in Section 3.2, and the optimization approach is based on particle swarm which will be described in Section 3.3. Translation parameter estimation is describe in details in Section 3.4.

### 3.1. Geometric Transformation

#### 3.1.1. Affine Transformation

In an affine transformation, the x and y dimensions can be scaled or sheared independently and there may be a translation, a reflection, and/or a rotation. Parallel lines remain parallel after affine transformation. The affine transformation can be expressed as:

(2) $$\left(\begin{array}{c} u\\ v \end{array}\right)\;=\;\left[\begin{array}{cc} a & b\\ c & d \end{array}\right]\left(\begin{array}{c} x\\ y \end{array}\right)+\left(\begin{array}{c} e\\ f \end{array}\right)$$

Here, we simplified the affine transformation as an affine rotation matrix *A* and a translation matrix *T*, as:

(3) $$\left(\begin{array}{c} u\\ v \end{array}\right)\;=\;A\left(\begin{array}{c} x\\ y \end{array}\right)+T$$

In the work of [30], the affine rotation matrix is determined by four affine parameters:

(4) $$A=\lambda \left[\begin{array}{cc} \cos\psi & -\sin\psi \\ \sin\psi & \cos\psi \end{array}\right]\left[\begin{array}{cc} t & 0\\ 0 & 1 \end{array}\right]\left[\begin{array}{cc} \cos\varphi & -\sin\varphi \\ \sin\varphi & \cos\varphi \end{array}\right]$$

where λ is the zoom parameter, ψ is the rotation angle of camera around optical axis, *t* links to the tilt angle θ between optical axis and normal to the image plane by $t\;=\;\frac{1}{\cos\theta }$ and φ is the longitude angle between optical axis and a fixed vertical plane.

Thus, the aim of image registration containing affine transformation is to search for the best A(λ,ψ,t,φ) which obtain the largest value of similarity through iterative optimization:

(5) $$A_{opt}:I_{1}\to I_{2}\;=\;maximize\left(S\left(I_{1}-A\left(I_{2}\right)\right)\right)$$

For two images taken from approximately same distance, the zooming factor λ can be set as 1 for quick optimization process. 

#### 3.1.2. Projective Transformation

Projective transformation enables the plane of the image to tilt, and thus parallel lines are not necessarily parallel and can converge towards a vanishing point. Affine transformation is a subset of perspective transformation. Projective transformation is a more general cases when match two images taken from different view angle, because the vanishing point creates the appearance of depth. The projective transformation can be expressed as a projective rotation matrix *P* and a translation matrix *T*, as:

(6) $$\left(\begin{array}{c} up\\ vp\\ wp \end{array}\right)\;=\;P\left(\begin{array}{c} x\\ y\\ w \end{array}\right)+T$$

Ideally, the projective matrix *P* is 3-by-3 matrix where all nine elements can be different, however, optimization for nine parameters will be time-consuming. A simplified projective matrix *P* is thus use in this paper by setting two free elements in the last column [31]:

(7) $$P\;=\;\left(\begin{array}{ccc} 1 & 0 & E\\ 0 & 1 & F\\ 0 & 0 & 1 \end{array}\right)$$

where *E* and *F* demotes the location of vanishing point in *x* and *y* direction. If *E* and *F* are large, the parallel lines appear to converge more quickly which means large perspective distortions.

The aim of optimization can be set as to search for the best P(E,F) which obtain the largest value of similarity:

(8) $$P_{opt}:I_{1}\to I_{2}\;=\;maximize\left(S\left(I_{1}-P\left(I_{2}\right)\right)\right)$$

### 3.2. Phase Correlation Based Simiarity Measure

Phase Correlation is an image matching algorithm based on the Fourier shift property, which states that a translation shift between two similar images generates a linear phase difference in the Fourier frequency domain [6]. In this paper, Phase Correlation has been used in two parts: (i) the similarity measure based on Dirac delta function; (ii) translation matrix estimation, which will be detailed described in Section 3.4.

Suppose that there is a translation shift (e,f) between two identical images $I_{1}\left(x,y\right)$ and $I_{2}\left(x,y\right)$:

(9) $$I_{1}\left(x,y\right)\;=\;I_{2}\left(x-e,y-f\right)$$

then, according to the shift properties shift property of the Fourier transform:

(10) $$F_{1}\left(u,v\right)\;=\;F_{2}\left(u,v\right)e^{-i\left(eu+fv\right)}$$

where $F_{1}\left(u,v\right)$ and $F_{2}\left(u,v\right)$ are Fourier transforms of the two images $I_{1}\left(x,y\right)$ and $I_{2}\left(x,y\right)$ Phase correlation, defined as the phase difference between $F_{1}\left(u,v\right)$ and $F_{2}\left(u,v\right)$, can be presented by a cross power spectrum Q(u,v):

(11) $$Q\left(u,v\right)\;=\;\frac{F_{1}\left(u,v\right)F_{2}{}^{*}\left(u,v\right)}{\left|F_{1}\left(u,v\right)F_{2}{}^{*}\left(u,v\right)\right|}\;=\;e^{-i\left(eu+fv\right)}$$

where * stands for complex conjugate.

Q(u,v) is a complex 2D matrix which can be presented by fringe, which density and orientation are $\surd \left(e^{2}+f^{2}\right)$ and e/f respectively. The inverse Fourier transform (IFT) of Q(u,v) is a Dirac delta function which its peak value $p_{\delta }$ ranges from 0 to 1:

(12) $$p_{\delta }\;=\;Max({\mathrm{FT}}^{-1}\left(Q\left(u,v\right)\right))$$

Obviously, if the two images are perfectly aligned, which means that the signals from two images are strongly correlated, there will be clear interference fringe pattern shown in Q(u,v) as shown in Figure 1, and the peak value $p_{\delta }$ will have a high value (close to 1). However, if two images contain large geometric distortions, the quality of interference fringes are deteriorated, so the peak value $p_{\delta }$ will have a relatively small value (close to 0). Thus, in this paper, the peak value of Dirac Delta function $p_{\delta }$ is use as similarity measure for the energy function in optimization.

### 3.3. Particle Swarm Optimization

Particle Swarm Optimization (PSO) [32], proposed by Kennedy and Eberhart, solves optimization problems by iteratively sampling candidate positions till an optimal measure of quality is achieved. It was intended for simulating social behavior, for example, foraging in a bird flock or fish school. PSO is initialized with a population of candidate solutions, called particles, $X_{i}=\left(x_{i1},x_{i2},\ldots ,x_{iN}\right)$. The particles are moved in the search space according to the rate of position change velocity $v_{id}$:

(13) $$x_{id}\;=\;x_{id}+v_{id}$$

At each time step, the position change velocity is determined towards its local best position $p_{best}$ and global best position $g_{best}$ according to the equation:

(14) $$v_{id}\;=\;wv_{id}+r_{1}c_{1}\left(p_{best}-x_{id}\right)+r_{2}c_{2}\left(g_{best}-x_{id}\right)$$

where w is inertial weight, $c_{1}$ is the self confidence factor adjust the weight of each particle’s best position when adjusting velocity, $c_{2}$ is the swarm confidence factor which adjust weight of the neighborhood’s best position when adjusting velocity, $r_{1}$ and $r_{2}$ are random numbers between [0,1]. 

In this paper, the fitness value is set as the Dirac delta peak value $p_{\delta }$, according to the Equation (2). The fitness value is with regards to geometric transformation parameters are energy function needs to be maximized. In affine transformation, the fitness value is $f\left(\lambda ,\psi ,t,\varphi ,p_{\delta }\right)$, where λ,ψ,t,φ are parameters from affine transformation and $p_{\delta }$ are Dirac delta peak values. Similarity, for projective transformation, the fitness value is $f\left(E,F,p_{\delta }\right)$, where E, F are parameters from projective transformation. As a heuristic optimization method, particle swarm can avoid the derivation of Dirac delta function, and thus has been applied to solve the geometric parameter optimization problems in this paper. The process of PSO is carried out as follows:

(1) Choose a population of particles with random initial positions and velocities
(2) The fitness value of each particle is calculated based on given geometric parameters
(3) Compare particle’s fitness value to its local best position. If current fitness value $f\left(x_{i}\right)$ is better than local best position $pbest_{i}$, then set local best position equal to current value
(4) Compare the fitness value with the neighborhood’s overall previous best. If the current value is larger than global best position gbest, then set global best position equal to current value
(5) Update the particle position *Xi* and velocity $V_{id}$ according to Equations (13) and (14)
(6) Loop to step (2) until the one of the criterions to stop the iteration is met.

### 3.4. Phase Correlation Based Translation Estimation

After the rotation transformation parameters $A_{opt}$ has been calculated from the PSO, the rotation between two images can be rectified. In this step, the translation matrix *T* will be estimated using Phase Correlation function PC by the target image $I_{1}$ and the warped image $A_{opt}\left(I_{2}\right)$:

(15) $$T\;=\;\left(\begin{array}{c} e\\ f \end{array}\right)\;=\;PC\left(I_{1},A_{opt}\left(I_{2}\right)\right)$$

As a non-iterative method, Phase Correlation can directly calculate the image shifts without roaming search. Moreover, phase correlation is able to achieve very high sub-pixel (1/100 sub-pixel) matching accuracies [33]. 

The shifts (e,f) can be estimated directly in the frequency domain or resolved in the spatial domain via IFT. The integer translation shift between two images can be estimated by the peak location of Dirac delta function $\delta_{0}$ that is the IFT of the cross power spectrum:

(16) $${\mathrm{FT}}^{-1}\left(Q\left(u,v\right)\right)\;=\;\delta_{0}\left(x-e,y-f\right)$$

Sub-pixel location can be determined by fitting a Gaussian function to the points which close to the peak of Dirac delta function $\delta_{0}$ [33]. The shift can also be resolved directly in the frequency domain with sub-pixel accuracy [17,18,19,20]. The cross power spectrum Q(u,v), which is a rank one matrix, and can be decomposed as the product of two dominant singular vectors $Q_{x}\left(e\right)$ and $Q_{y}\left(f\right)$:

(17) $$Q_{2}\;=\;e^{-i\left(ue+vf\right)}\;=\;e^{-iue}e^{-ivf}\;=\;Q_{x}\left(e\right)Q_{y}\left(f\right)$$

The sub-pixel displacement can then be estimated directly in the frequency domain using either Singular Value Decomposition (SVD) and then Least Square Fitting (LSF) [17], or 2D fitting technique with Quick Maximum Density Power Estimator (QMDPE) [19,34]. The frequency-based PC sub-pixel estimation method is able to achieve higher matching accuracy than spatial-domain based PC method, and thus, in this paper, SVD-PC is used to estimate the translation matrix T.

## 4. Experiments and Discussion

### 4.1. Dataset

**Figure 3:**
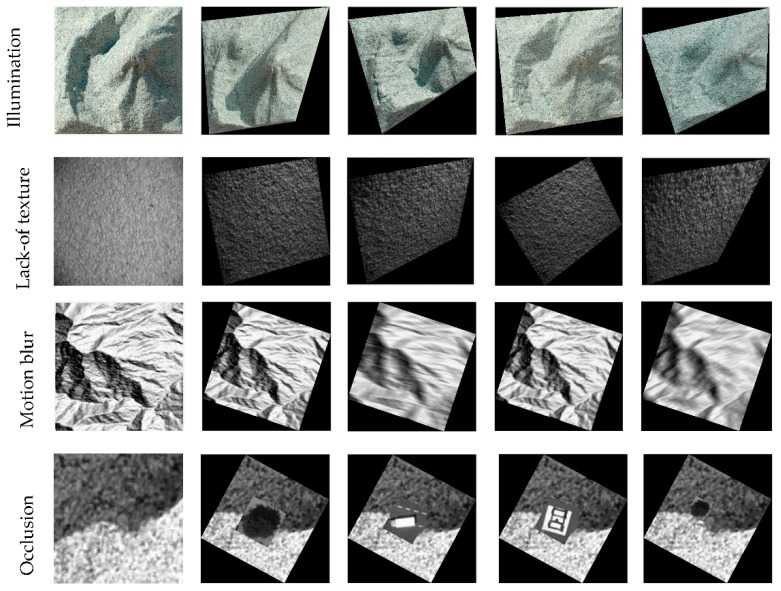
Image dataset which containing illumination variation, lack-of-texture, motion blur and occlusion.

**Figure 4:**
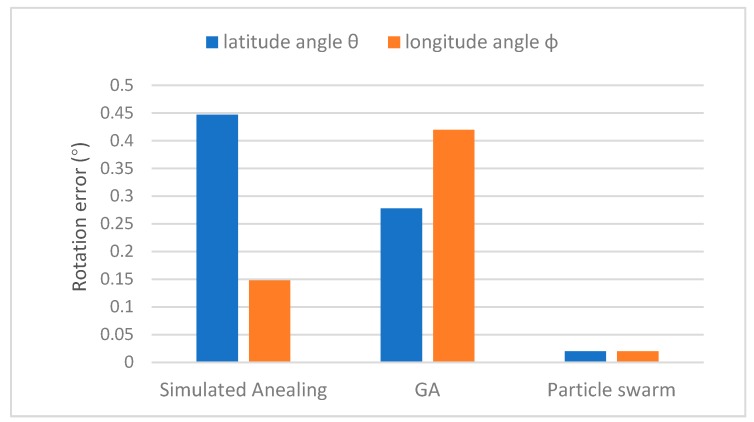
Transformation rotation errors by three optimization algorithms: simulated annealing, GA and particle swarm.

Image registration tasks are carried out using image pairs which contain challenges including illumination variation, lack of texture, motion blur, occlusion and different geometric distortions, as shown in Figure 3. For the illumination image pairs, 10 images from the same position are taken from 9:30 to 15:30 on a terrain model under daily sunlight variation, and then the image taken in 10:30 is used as target image, and the remaining nine images are warped by perspective transformation and use as reference image, as shown in the first row in Figure 3. The lack-of texture dataset is downloaded from the webpage of TextureLab at Heriot-Watt University. This dataset includes images from different texture surfaces. The light source in this dataset is a fixed desk-lamp (+12 V, max 0.45 A) and a Vosskuhler CCD 1300LN digital camera is used to take images. Here we selected 10 pairs of textureless image pairs, and performed perspective transformation on the reference image, which are shown in the second row of Figure 4. 

### 4.2. Opmization Approach Comparison

Firstly, we compare our optimization method, particle swarm, with two state-of-the-art optimization methods, simulated annealing and Genetic Algorithm (GA). Both simulated annealing and GA are non-derivative-based optimization methods which all start with initial candidate positions and generate a population of points at each iteration. 

In the comparison experiment, we use the dataset described in Section 4.1 and apply affine transformations to the tested dataset and calculate the accuracies of the parameters estimated by different optimization methods. As the rotation matrix is determined from optimization, we focused on two rotation angles, latitude angles θ and longitude angles φ, in this experiment. For each image pair, we apply 30 latitude angles θ and 30 longitude angles φ ranges from 0° to 30° to the reference image, and the optimization accuracy is evaluated by the difference between the calculated angles and the ground truth.

The empirical optimization settings are shown in Table 2, where several parameters in common are the same for the three optimization methods for fair comparison. The maximum iteration and function tolerance are parameters to stop the iteration. A large maximum iteration and small function tolerance indicate strict constraint for optimization, which means more accurate result with more iterations. To balance the precision and speed of optimization, in this paper, maximum iteration and function tolerance are set as 10,000 and $1\times 10^{-9}$. The low and upper bounds are minimum and maximum values of the estimated angles. In this paper, as the ground truth values of latitude and longitude angles range from 0° to 30°, the low and upper bounds are set as 0 and 50. The number of swarm/chromosomes in PSO and GA is set as 15. A large swarm size value can avoid the optimization from falling into as local optimum, but will certainly increase the time required for optimization. For Simulated Annealing, the initial temperature is set as 5000 and a slow cooling factor 0.95 is set to allow the temperature to go down slowly at first but ultimately get cooler faster until converge to the optimal solution. For PSO, the inertial weighting factor w is set as 0.729 under Clerc’s constriction factor [35], while both self confidence factor and swarm confidence factor are set to 1.49, also according to Clerc’s constriction factor. Setting $c_{1}$ and $c_{2}$ equal can avoid the optimization stuck in current optima. The rotation transformation errors calculated from three optimization approaches are shown in Figure 4.

As shown in Figure 4, the rotation angle errors estimated by simulated annealing and GA are approximately 0.3°, which are acceptable for general image registration cases where a precise rotation angle is not required. Particle swarm-based optimization, in comparison, achieves higher accuracy (0.02°) compared to the two state-of-the-art methods. Compared to simulated annealing and GA, the velocity of particles in PSO are randomly initialized and optimized, which enables the optimization to quickly converge to a global optimum. Moreover, in each iteration, the particles can ‘learn’ from previous positions and other particles via fitness functions, and thus higher accuracies can be determined based on this self-learning strategy. The experimental results demonstrate that particle swarm optimization-based registration can achieve accurate estimations of rotation angles between two images and thus can be used for registration cases which require high rotation angle accuracy. 

### 4.3. Robustness with Respect to Different Geometric Distortions

In this section, we tested the robustness of the proposed method towards affine and perspective distortions in mutiple runs. For affine distortions, the tilt angle $\theta_{i}$ and longitude angle $\varphi_{i}$ are varying from 0° to 89° with interval of 5°, so a number of affine transformations with different distorted parameters are generated $A\left(\theta_{i},\varphi_{i}\right)$. Then, image registration is carried out using the initial image $I_{1}$ and the distorted image $A\left(I_{1}\right)$ which results in the calculated tilt angle $\theta_{i}{}^{*}$ and longitude angle $\varphi_{i}{}^{*}$. The registration accuracies are evaluated by the error between calculated and groundtruth tilt angle and longitude angle:

(18) $$tilt\;error\;=\;|\theta_{i}{}^{*}-\theta_{i}|\;longitude\;error\;=\;|\varphi_{i}{}^{*}-\varphi_{i}|$$

**Figure 5:**
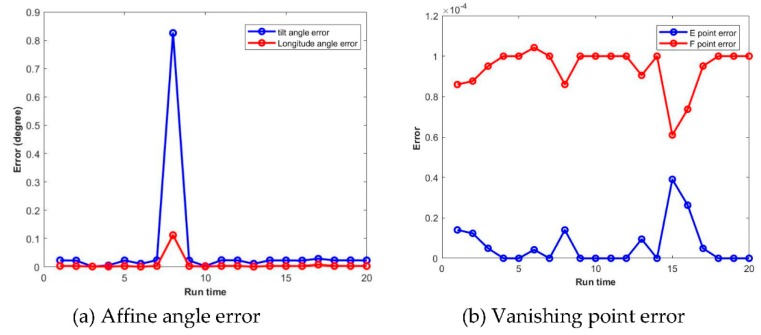
Matching error with respect to affine angle and vanishing point variations in 20 runs. (**a**) Affine angle error. (**b**) Vanishing point error.

For each input pair of longitude and tilt angle, 20 runs were used to test the repeatability of the proposed method. One example of multiple runs for affine angle estimation is shown in Figure 5a. As the initial positions of particles are randomly determined, the particle swarm optimization may show sligntly different result from the same image input, and thus the image matching accuracy may have some variations, as shown in Figure 5a. For example, during the 8th run, the tilt error is as large as 0.8°, which is larger than in the other runs. This image matching failure case is likely due to the the situation that particles fall into local minimum rather than global minumum in optimization. The final affine angle error is evaluated by mean and standard deviation errors as shown in Table 3. Although the results have fluctuations, according to Table 3, the average tilt error is 0.05° and the average longitude error is 0.008°.

Similarly, for perspective distortion, a series of $E_{i}$ and $F_{i}$ are given to simulate different perspective transformations $P\left(E_{i},F_{i}\right)$. The registration accuracies are evaluated by the error between calculated and groundtruth *E* and *F*, and one multiple run result is shown in Figure 5b. The mean and standard deviation of perspective transformation estimation error are listed in Table 3. Similar to the case in affine angle variation, the image matching error did show randomness, but the mean and standard deviation demonstrate the high average accuracies of the proposed method. In the latter section, to reduce the randomness of the PSO, the image matching results are based on 20 runs.

### 4.4. Similarity Measure Comparison

In this section, we compare phase correlation as similarity measure with respect to two state-of-the-art similarity measures: NCC and MI. The registration accuracies is evaluated as follows: 12 control points are selected to uniformly distributed on the target images, so their groundtruth positions $\left(\hat{u},\hat{v}\right)$ after performed geometric transformation can be determined $\left(\hat{u},\hat{v}\right)\;=\;\hat{G}\left(x,y\right)$, where (x,y) are the original position of the control points, and $\hat{G}$ denoted as the geometric transformation. After registration, the calculated positions of the control points can be determined as (u,v) = G(x,y), where the transformation matric G is calculated from image registration. The image registration accuracies is assessed by the absolute difference between groundtruth and calculated positions of 12 control points:

(19) $$\sigma \;=\;\left(\left|\hat{u}-u\right|+\left|\hat{v}-v\right|\right)/2$$

**Figure 6:**
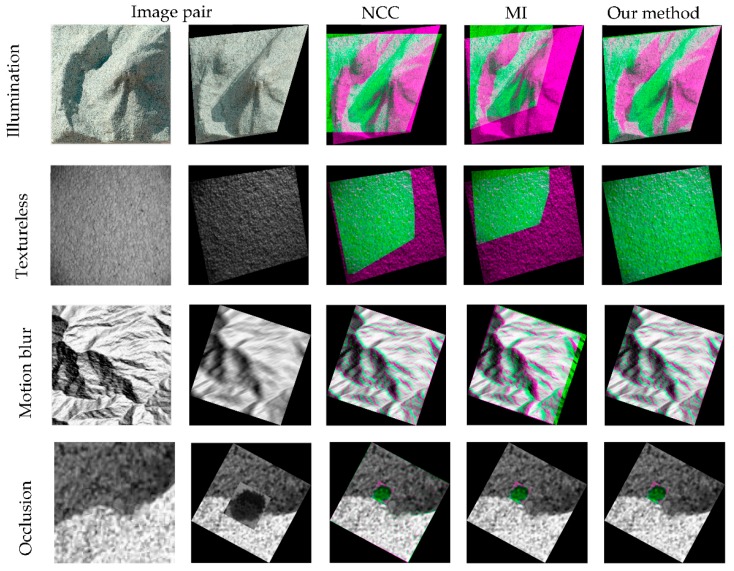
Image registration comparison between different similarity measures.

Some of the image registration results using NCC, MI and phase correlation, which used in this paper, are shown in Figure 6. The average registration accuracies using the three similarity measures are shown in Table 4. 

The experiment results in Figure 6 demonstrate that, compared to NCC and MI phase correlation is more robust in the cases of illumination variation and lack of texture, with sub-pixel registration accuracy of 0.57 pixel and 0.065 pixel, respectively, while NCC and MI all fail in the two cases. All three measures are able to cope with motion blur and occlusion, but NCC and phase correlation reach higher registration accuracy (0.168 pixel and 0.012 pixel) compared to others. As a frequency-based image matching method, Phase Correlation is able to identify the topography and texture similarity between image pairs regardless of illumination variation. As the image translation is calculated by phase difference, which is estimated in the frequency domain, high registration accuracies can be achieved by transforming the phase difference into the spatial domain. According to Table 4, the overall average registration accuracy of phase correlation is approximately 0.3 pixel considering four challenges. The experiment results demonstrate that frequency-based similarity measures are more robust to illumination variation and lack of texture cases, and achieve higher registration accuracies when all similarity measures succeed.

### 4.5. Multimodal Image-Based Navigation

In this section, we demonstrate one of the possible applications using the proposed registration method: image-based navigation. According to the definition of cross power spectrum, phase correlation is able to align two multimodal images as long as they are highly similar in the frequency domain. In this experiment, the robustness of the proposed method with respect to multimodal image registration will be demonstrated by locating the optical images using a DEM as reference data. 

**Figure 7:**
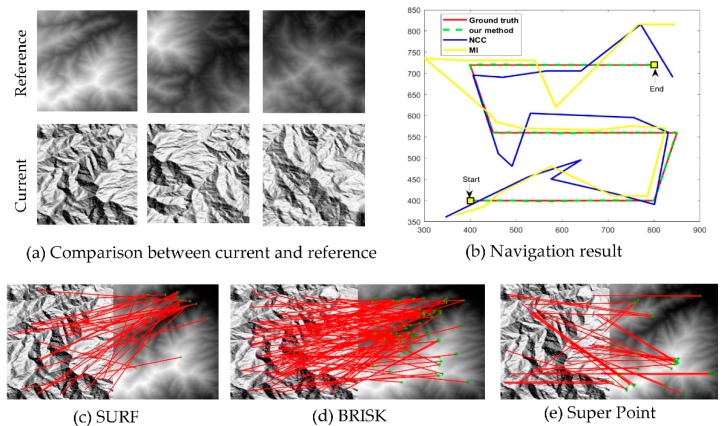
DEM referenced image-based navigation. (**a**) shows the input current and reference images, (**b**) shows the navigation results of proposed methods in comparison with NCC and MI, (**c**,**d**,**f**) demonstrate the image matching results of SURF, BRISK and Super Point respectively.

Here, a birdview sequence is generated from a reference DEM given certain lighting conditions and perspective viewing angle. The goal is to recover the trajectory of camera by the image alignment between the birdview image and the reference image. The challenge in this experiment is that the birdview image and reference image (DEM) are from different modalities, so the image texture can be very different, as shown in Figure 7a. 

To balance the matching accuracy and processing time, five optimization runs are used in this experiment. The trajectories estimated by state-of-the-art methods and our method are shown in Figure 7b. Some of the feature-based image matching results are shown in Figure 7c–f. The navigation accuracies are assessed by the difference between ground truth and calculated positions in the *x* and *y* directions, respectively, and shown in Table 5.

As shown in Figure 7 and Table 5, feature based methods, such as SURF, BRISK and Super Point fail to perform the DEM-based navigation owing to the large appearance difference between DEM and the optic image. As correlation-based methods, NCC and MI perform slightly better than feature-based methods, but they also fail to recover the correct trajectory with large deviations from the ground truth. In comparison, the proposed PC-based registration is able to correctly estimate the camera trajectory with registration errors within 1 pixel. This experiment demonstrates that frequency-based similarity measures are more robust to multimodal images than spatial-based measures, such as NCC and MI. Although the reference and target images in this experiment contain large appearance differences, they are intrinsically derived from the same terrain model and share enough information which could be correlated in the frequency domain.

## 5. Conclusions

In this paper, we extended phase correlation image matching from Euclidean transformation to perspective and affine geometric transformation by combining with the particle swarm optimization method. The qualities of fringes in the phase correlation cross power spectrum are used as similarity measures, and the aim of optimization can be set as to search for the best transformation parameters which obtain the largest value of similarity. Image registration experiments demonstrated that compared to state-of-the-art image registration methods, only the proposed method can cope with all challenges, including illumination, lack-of texture, motion blur and occlusion and different geometric distortions and reach the image registration accuracies within 1 pixel. Further, image-based navigation experiments are carried out demonstrated that the proposed method is able to correctly recover the trajectory of camera using multimodal target and reference images.

In this paper, the transformation matrix is set as affine and perspective, but it could be easily further applied to other distortions, such as polynomial transformations, which are widely used in satellite image registration. Future work will be focused on the combination of reference-based navigation and visual odometry to ensure the speed and accuracy of the navigation.

## Figures and Tables

**Table 1:** The robustness of matching approaches towards radiometric and geometric distortions.

	Radiometric Distortion		Geometric Distortions	
	Lack-of Texture	Multimodal	Euclidean	Perspective
Feature	No	Limited	Yes	Yes
Correlation	Yes	Yes	Yes	No
Ideal	Yes	Yes	Yes	Yes

**Table 2:** Parameter setting for optimization.

Simulated Annealing
Initial temperature: 5000 Cooling factor: 0.95
**PSO**
w=0.729 $c_{1}=1.49$ $c_{2}=1.49$
Maximum iteration: 1 $\times \;10^{4}$ Function tolerance: $1\;\times \;10^{-9}$ Number of chromosomes/particles: 15 Low bound: 0 upper bound: 50

**Table 3:** Mean and standard deviation of the proposed methods under affine and perspective transformation.

Error	Affine Transformation		Perspective Transformation	
	Longitude	Tilt	*E*	*F*
Mean	0.0084	0.0596	6.46 $\times 10^{-6}$	9.35 $\times 10^{-5}$
Standard deviation	0.0079	0.0245	1.04 $\times 10^{-5}$	1.07 $\times 10^{-5}$

**Table 4:** Image registration accuracies using different similarity measures (pixel).

Algorithms	Illumination + Perspective	Lack of Texture + Perspective	Blur + Affine	Occlusion + Affine
NCC	32.272	26.913	**0.168**	1.804
MI	35.821	45.034	0.884	0.588
Our method	**0.570**	**0.065**	0.655	**0.012**

Bold shows the best performance.

**Table 5:** Image-based navigation accuracies using different similarity measures (pixel).

Algorithms	NCC	MI	SURF	Super Point	BRISK	Our Method
*x*	48.111	31.666	46	70	100.86	**0.981**
*y*	33.722	40.277	139	93	102.82	**0.648**

Bold number demonstrate the best performance.

## References

[1] Zhang Z. (2000). A flexible new technique for camera calibration. IEEE Trans. Pattern Anal. Mach. Intell..

[2] Pons J.-P., Keriven R., Faugeras O. (2007). Multi-view stereo reconstruction and scene flow estimation with a global image-based matching score. Int. J. Comput. Vis..

[3] Gaspar J., Winters N., Santos-Victor J. (2000). Vision-based navigation and environmental representations with an omnidirectional camera. IEEE Trans. Robot. Autom..

[4] Irani M., Peleg S. (1991). Improving resolution by image registration. CVGIP Graph. Models Image Process..

[5] Gonzalez R. Improving Phase Correlation for Image Registration. Proceedings of the Image and Vision Computing New Zealand.

[6] Kuglin C. The Phase Correlation Image Alignment Methed. Proceedings of the International Conference Cybernetics Society.

[7] Foroosh H., Zerubia J.B., Berthod M. (2002). Extension of phase correlation to subpixel registration. IEEE Trans. Image Process..

[8] Canny J. (1986). A computational approach to edge detection. IEEE Trans. Pattern Anal. Mach. Intell..

[9] Haralick R.M. (1984). Digital step edges from zero crossing of second directional derivatives. Pattern Anal. Mach. Intell. IEEE Trans..

[10] Marr D., Hildreth E. (1980). Theory of edge detection. Proc. Royal Soc. Lond. Ser. B Biol. Sci..

[11] Lowe D.G. (2004). Distinctive image features from scale-invariant keypoints. Int. J. Comput. Vis..

[12] Bay H., Tuytelaars T., Van Gool L. (2006). Surf: Speeded up Robust Features.

[13] Verdie Y., Yi K., Fua P., Lepetit V. TILDE: A temporally invariant learned detector. Proceedings of the IEEE Conference on Computer Vision and Pattern Recognition.

[14] Yi K.M., Trulls E., Lepetit V., Fua P. Lift: Learned invariant feature transform. Proceedings of the European Conference on Computer Vision.

[15] DeTone D., Malisiewicz T., Rabinovich A. Superpoint: Self-supervised interest point detection and description. Proceedings of the IEEE Conference on Computer Vision and Pattern Recognition Workshops.

[16] Papoulis A., Pillai S.U. (2002). Probability, Random Variables and Stochastic Processes with Errata Sheet.

[17] Hoge W. (2003). A subspace identification extension to the phase correlation method. IEEE Trans. Med Imaging.

[18] Hoge W.S., Westin C.F. (2005). Identification of translational displacements between N-dimensional data sets using the high-order SVD and phase correlation. IEEE Trans. Image Process..

[19] Liu J.G., Yan H. (2008). Phase correlation pixel-to-pixel image co-registration based on optical flow and median shift propagation. Int. J. Remote Sens..

[20] Morgan G.L.K., Liu J.G., Yan H. (2010). Precise subpixel disparity measurement from very narrow baseline stereo. IEEE Trans. Geosci. Remote Sens..

[21] Reddy B.S., Chatterji B. (1996). An FFT-based technique for translation, rotation, and scale-invariant image registration. IEEE Trans. Image Process..

[22] Wan X., Liu J.G., Yan H. (2015). The Illumination Robustness of Phase Correlation for Image Alignment. IEEE Trans. Geosci. Remote Sens..

[23] Zitova B., Flusser J. (2003). Image registration methods: A survey. Image Vis. Comput..

[24] Effendi S., Jarvis R. Camera Ego-Motion Estimation Using Phase Correlation under Planar Motion Constraint. Proceedings of the 2010 International Conference on Digital Image Computing: Techniques and Applications.

[25] Keller Y., Averbuch A. (2007). A projection-based extension to phase correlation image alignment. Signal Process..

[26] Barnada M., Conrad C., Bradler H., Ochs M., Mester R. Estimation of automotive pitch, yaw, and roll using enhanced phase correlation on multiple far-field windows. Proceedings of the Intelligent Vehicles Symposium (IV).

[27] Song G., Han J., Zhao Y., Wang Z., Du H. (2017). A review on medical image registration as an optimization problem. Curr. Med Imaging Rev..

[28] So R.W., Tang T.W., Chung A.C.A. (2011). Non-rigid image registration of brain magnetic resonance images using graph-cuts. Pattern Recognit..

[29] Jia D.L.F. A Comparison of Simulated Annealing, Genetic Algorithm and Particle Swarm Optimization in Optimal First-order Design of Indoor TLS Networks. Proceedings of the ISPRS Geospatial Week Wuhan.

[30] Yu G., Morel J.-M. (2011). ASIFT: An algorithm for fully affine invariant comparison. Image Process. Line.

[31] Beutelspacher A., Rosenbaum U. (1998). Projective Geometry: From Foundations to Applications.

[32] Kennedy J. (2010). Particle swarm optimization. Encycl. Mach. Learn..

[33] Argyriou V., Vlachos T. A Study of Sub-pixel Motion Estimation using Phase Correlation. Proceedings of the BMVC.

[34] Yan H., Liu J. Robust Phase Correlation based Motion Estimation and Its Applications. Proceedings of the British Machine Vision Conference.

[35] Clerc M. The swarm and the queen: Towards a deterministic and adaptive particle swarm optimization. Proceedings of the 1999 Congress on Evolutionary Computation.

